# The efficacy of the traditional Chinese medicine Juanbi pill combined with methotrexate in active rheumatoid arthritis: study protocol for a randomized controlled trial

**DOI:** 10.1186/s13063-018-2555-1

**Published:** 2018-03-20

**Authors:** Qiong Wang, Yi-Ru Wang, Qing-Yun Jia, Li Liu, Chong-Qing Xu, Xiao-Yun Wang, Min Yao, Xue-Jun Cui, Qi Shi, Yong-Jun Wang, Qian-Qian Liang

**Affiliations:** 1 Department of Orthopaedics, Longhua Hospital, Shanghai University of Traditional Chinese Medicine, 725 South Wan-Ping Road, Shanghai, 200032 China; 2 Institute of Spine, Shanghai University of Traditional Chinese Medicine, 725 South Wan-Ping Road, Shanghai, 200032 China; 3 Rehabilitation Medicine College, Shanghai University of Traditional Chinese Medicine, 1200 Cai Lun Road, Shanghai, 201203 China; 4 Key Laboratory of theory and therapy of muscles and bones, Ministry of Education Shanghai University of Traditional Chinese Medicine, Shanghai, China

**Keywords:** Traditional Chinese medicine, Juanbi pill, Methotrexate, Active rheumatoid arthritis, Randomized controlled trial

## Abstract

**Background:**

Rheumatoid arthritis (RA) is a chronic autoimmune disease characterized by swelling and painful joints, eventually leading to joint destruction. There is still a lack of effective therapy to treat RA. The Juanbi pill is a Chinese medicine that has been widely used to treat active RA in China for hundreds of years, relieving pain and protecting the affected joints from malformation. However, there is no solid evidence to show the effect of the Juanbi pill on the management of active RA.

**Methods/design:**

We will conduct a multicenter, randomized, double-blind, placebo-controlled clinical trial to determine whether the traditional Chinese medicine Juanbi pill could relieve joint pain in RA and protect the joints. A total of 120 patients with active RA will be enrolled and treated with the Juanbi pill or a placebo for 3 months. The primary outcome measures are as follows: rate of in the American College of Rheumatology (ACR)50, change in the 28-joint Disease Activity Score (DAS28) from baseline at beginning of therapy to 3 months, and a change in the van der Heijde modified Sharp score measured from baseline to 12 months. The secondary outcome measures are as follows: rate of change in ACR20, ACR70, Health Assessment Questionnaire-Disability Index (HAQ-DI), and change in score in the Patient Assessment of Arthritis Pain, Patient Global Assessment of Arthritis, and the Athens Insomnia Scale (AIS) from baseline to 2-week, 1-month, 2-month, 3-month, 6-month, and 12-month follow up. In addition, the rate of change (score) in the ACR50 and DAS28 from the baseline to 2-week, 1-month, 2-month, 6-month, and 12-month follow up are also the secondary outcome measures.

**Discussion:**

Although the Juanbi pill has been used in China for many years to treat RA, there is a lack of consensus about its effectiveness. This trial will provide convincing evidence about the effect of Juanbi pill on active RA.

**Trial registration:**

ClinicalTrials.gov, NCT02885597. Registered on 30 August 2016.

**Electronic supplementary material:**

The online version of this article (10.1186/s13063-018-2555-1) contains supplementary material, which is available to authorized users.

## Background

Rheumatoid arthritis (RA) is a chronic autoimmune disease that is characterized by pain, swelling, synovial inflammation, joint damage, and bone destruction, resulting in severe disability and increased mortality rate [1]. The prevalence of RA worldwide is estimated to be 0.5–1%, with a mean annual incidence of 0.05% [2, 3]. Much research has been done to find an approach to alleviate the swelling and tenderness of joints and avoid irreversible joint impairment. Nevertheless, the treatment approaches for RA are still limited [4].

Herbal medicines, which are complementary and alternative medicines (CAM), have been used for centuries in China to treat different illnesses [5, 6]. In traditional Chinese medicine (TCM), RA is known as *Bi syndrome*, and *Juan Bi* (means getting rid of *Bi syndrome* in Chinese) decoction from the book *Yi Xue Xin Wu* from the Qing Dynasty was specially designed to treat *Bi syndrome* [7]. The Juanbi decoction is composed of Radix angelicae pubescentis, Notopterygium incisum, *Cinnamomum cassia* Presl, Gentiana macrophylla Pall., Angelica sinensis (Oliv.) Diels, Ligusticum chuanxiong Hort., Glycyrrhiza uralensis Fisch., Caulis Piperis Kadsurae, *Morus alba* L, Olibanum, and Radix Aucklandiae, and has been widely used to treat arthritis in China [8–10]. Our preliminary screening identified that Juanbi decoction attenuates inflammation of the ankle joints in TNF-transgenic (TNF-Tg) mice, a chronic inflammatory arthritis mouse model (data not shown).

Juanbi decoction combined with methotrexate (MTX) has been widely used in China for treatment of RA, and case report studies show that Juanbi decoction can help MTX attenuate joint pain and swelling [11–13]. However, there are few data from large-scale randomized trials on the efficacy of Juanbi for the treatment of RA and its adverse effects. Therefore, we aimed to conduct a randomized, double-blinded, placebo-controlled trial to estimate the effectiveness and safety of Juanbi decoction for RA. The results of this trial will provide evidence on the value of the Juanbi decoction as an intervention to lower disease activity in RA and protect the affected joints from deformity. Considering the difficulty in assessing the quality of a decoction, we instead plan to use the Juanbi pill in our trial. The Juanbi pill is the raw extraction of Juanbi decoction used for convenience of quality control and long-term storage, and it has been used in our hospital for decades.

## Methods/design

### Study design

**Fig. 1:**
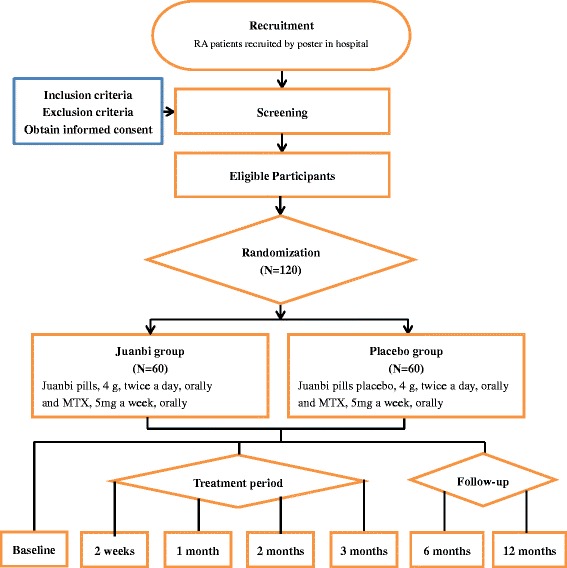
Project overview. RA, rheumatoid arthritis; MTX, methotrexate

This study is a multicenter, randomized, double-blinded, placebo-controlled clinical trial with two parallel arms (Fig. 1). The aim of the study is to evaluate whether the Juanbi pill combined with MTX is effective for the management of active RA. In total, 120 patients with active RA will be recruited (60 patients per arm) from centers in Shanghai, China, from Longhua Hospital affiliated with Shanghai university of Traditional Chinese Medicine, Yueyang Integrated Medicine Hospital affiliated with Shanghai University of Traditional Chinese Medicine, and Shanghai Guanghua Hospital of Integrated Traditional Chinese and Western Medicine. The Standard Protocol Items: Recommendation for Interventional Trials (SPIRIT) 2013 checklist is shown in Additional file 1.

### Study participants

Participants will be recruited from three hospitals (Longhua Hospital, Shanghai Yueyang Integrated Medicine Hospital, and Shanghai Guanghua Hospital of Integrated Traditional Chinese and Western Medicine) by poster and hospital official website advertisements. The planned recruitment period is 12 months. Informed consent will be obtained from all participants before randomization.

### Eligibility criteria

#### Inclusion criteria

Participants who fulfill the following criteria are eligible:

- Adults (aged ≥ 18 y) with RA
- Satisfy American College of Rheumatology (ACR)/European League Against Rheumatism (EULAR) criteria for RA, 2010 [4]
- Onset of symptoms within 12 months before enrollment
- Active disease at the time of enrollment as indicated by 28-joint Disease Activity Score (DAS28) greater than 3.2 [14], and no prior exposure to oral glucocorticoids at a daily dose greater than 10 mg or to any biologic agents
- Paid or unpaid employment but measurable work (e.g., caring for a family and home)

#### Exclusion criteria

The exclusion criteria are as follows:

- RA combined with other autoimmune disease, such as adjuvant arthritis, lupus arthritis, or osteoarthritis
- RA combined with abnormal liver and kidney function
- Women who are pregnant, planning pregnancy, or breast-feeding
- Severe chronic or acute disease interfering with attendance for therapy
- Alcohol or substance abuse
- Unable to understand or sign an informed consent form

### Interventions

All patients will be randomized into the Juanbi group or the placebo group. Patients in the Juanbi group will receive both the Juanbi pill (4 g/bag, two bags per day) and MTX (5 mg per week) for 3 months, whereas patients in the other group will receive a Juanbi pill placebo (4 g/bag, two bags per day) and the same dose of MTX as the Juanbi group. The Juanbi pill and the placebo are to be dissolved in 200 mL of hot water and taken orally twice a day for 3 months. The Juanbi pill will be manufactured, packaged, and labeled by Jiangyin Tianjiang Pharmaceutical Co., Ltd., based on good manufacturing practice standard. The crude herbs, including Rhizoma Et Radix Notopterygii (Qiang Huo) 18 kg, Radix Angelicae Pubescentis (Du Huo) 18 kg, Radix Gentianae Macrophyllae (Qin Jiao) 18 kg, Caulis Piperis Kadsurae (Hai Feng Teng) 36 kg, Ramulus Mori (Sang Zhi) 54 kg, Radix Angelicae Sinensis (Dang Gui) 54 kg, Rhizoma Chuanxiong (Chuan Xiong) 12.6 kg, Olibanum (Ru Xiang) 25.2 kg, Radix Aucklandiae (Mu Xiang) 25.2 kg, Radix Glycyrrhizae (Gan Cao) 9 kg, and Ramulus Cinnamomi (Gui Zhi) 9 kg, were supplied in one batch from Haozhou Hongyu Chinese Herbal Medicine Co., Ltd. or Anhui Huizhongzhou Chinese Herbal Medicine Co., Ltd. The test results of all herbs meet the standard. All herbs were stored in a specialized cool and dry place. The Juanbi pill is made as follows: (1) extraction: all herbs listed are put in a ceramic container and 1000 L of distilled water is added to macerate the herbs for 1 h. Then, the mixture is boiled at 100 °C for 1 h for the first extraction, and repeated twice to obtain three extractions in total. Note that the third extraction should contain only 500 L of distilled water and have only 30 min boiling time; (2) concentration: the three extractions are mixed and the mixture is concentrated at 60 °C (660 mmHg). The mixture is sprayed dry to produce an extract powder, which is smashed and screened through mesh size 80. The pills are packed in quantities of 4 g per bag and stored in a clean room at approximately 20 °C and 50% humidity. The Juanbi pill placebo contains 10% Juanbi pill and 90% bitterant, lactose edible essence, pigment (e.g., lemon yellow, caramel pigment, or sunset yellow), and starch, and has a similar shape, smell, color, and taste to the actual Juanbi pill. The Juanbi pill and the placebo all met the quality inspection standards.

The duration of the intervention is 3 months, and follow up is 9 months. Study visits will take place at baseline and at 2, 4, 8, 12, 24, and 52 weeks. Every patient will be asked to visit within 3 days of the given time point.

### Randomization and allocation

Randomization will be conducted by a credible pharmaceutical manufacturer (Jiangyin Tianjiang Pharmaceutical Co., Ltd), which will provide both the Juanbi pill and the placebo. A random number list will be generated using Excel (Office 2016, Microsoft Corporation) to randomize participants either to the Juanbi pill group or the placebo group in a 1:1 ratio. When a participant is recruited, the investigator will provide the pharmaceutical manufacturer with a number, and the pharmaceutical manufacturer will randomly post the Juanbi pill or the placebo to the participant according to the random number list. The number provided and the corresponding number list will be recorded and will be kept in a locked cabinet at the pharmaceutical company.

### Blinding

The pharmaceutical company staff will not participate in the trials, and the investigator, physicians, nurses, outcome measurement operative, statisticians, and the participants will be blinded to the group information until the end of the trial, when all statistical analyses are finished.

### Outcome measures

#### Primary outcome measure

The primary outcome measure will be as follows: (1) the progression of ACR 50 after 1, 2, and 3 months of treatment compared to baseline; (2) the change in DAS28 before and after 1, 2, and 3 months of treatment; and (3) the difference between the two groups in the van der Heijde modified Sharp score before and after 12 months of follow up.

The ACR50 is a scale to measure change in RA symptoms [15, 16]. The ACR50 requires the following: 50% or greater improvement in tender joint count, 50% or greater improvement in swollen joint count, and at least 50% improvement in three of the following five measurements: (1) assessment of the patient’s arthritis pain using a visual analogue scale (VAS) of 0–100 mm, (2) global assessment of the patient’s disease activity using a VAS (0–10), (3) assessment of the patient’s physical function and disability using the HAQ-DI, (4) an acute-phase reactant value such as erythrocyte sedimentation rate (ESR), or (5) C-reactive protein (CRP) level. ACR20 and ACR70 (measured as ≥ 20% improvement and ≥ 70% improvement, respectively) are required in the tender joint count, swollen joint count, and in 3 of the other 5 measurements [16].

Although compared to ACR50 and ACR70, ACR20 has been accepted as the efficacy benchmark in RA clinical trials and has greater discriminant capacity to distinguish patients on active treatment from placebo control [15, 17, 18], we choose ACR50 as the primary outcome measure because ACR50 is a more desirable target for patients and provides useful information in addition to ACR70 [19, 20].

The DAS28 is widely used as an indicator of RA disease activity and response to treatment, and clinical trials have used DAS28 to assess treatment effect [14, 21]. The formula for DAS 28 is:

$$ 0.56\times \sqrt{28\ \mathrm{painful}\ \mathrm{joint}\ \mathrm{count}}+0.28\times \sqrt{28\ \mathrm{swollen}\ \mathrm{joint}\ \mathrm{count}}+0.70\times \left(\mathrm{in}\ \mathrm{ESR}\right)+0.014\times \mathrm{GH}. $$

The 28 joints are 10 proximal interphalangeal joints and 10 proximal interphalangeal joints of the hands and wrists, elbows, knees, and shoulders bilaterally. GH is patient general health measured by VAS (0–100 mm); “0” is the best and “100” is the worst [16, 22–24].

The van der Heijde modified Sharp score is the radiographic assessment of the *erosion* and *the joint narrowing* in the 10 metacarpophalangeal joints, 8 proximal interphalangeal joints, 10 metatarsophalangeal joints, and 2 interphalangeal joints of the big toes. In addition, erosion is also assessed in the two interphalangeal joints of the thumbs, first metacarpal bone, radius, ulnar bones, trapezium and trapezoid, navicular bones, and right and left lunate bones bilaterally, whereas joint narrowing is also assessed in the third, fourth, and fifth carpometacarpal joints, multiangular-navicular joints, capitate-navicular-lunate joints, and right and left radiocarpal joints bilaterally. The maximum erosion score in the hand is 5, and in the foot it is 10, according to the degree of erosion. Thus, the maximum number of erosions in the hand is 160, and in the feet it is 120. The grade of joint space narrowing is as follows:

- 1 = focal or doubtful
- 2 = general, less than 50% of the original joint space
- 3 = general, more than 50% of the original joint space or subluxation
- 4 = no joint space remaining, luxation, or ankylosis

Therefore, the maximum score for joint space narrowing is 120 in the hand and 48 in the feet. The van der Heijde modified Sharp score is the sum of the erosion and joint space narrowing score [16, 25].

The ACR50 and DAS28 will be estimated at baseline and after 1, 2, and 3 months of treatment, and at the 6-month and 12-month follow up. The van der Heijde modified Sharp score will be measured only at baseline and after 12 months.

#### Secondary outcome measures

The secondary outcome will be to compare the rate of change in the ACR20/70, HAQ-DI, Patient Assessment of Arthritis Pain, Patient Global Assessment of Arthritis, and AIS from baseline to 2 weeks, 1 month, 2 months, 3 months, and at the 6-month and 12-month follow up. The rate of change in the ACR50 and DAS28 from t baseline to 2 weeks, 1 month, 2 months, and 6-month and 12-month follow up are also secondary outcome measures, and the change in score on the 36-item Short-Form Health Survey Questionnaire (SF-36) from baseline to 1 month, 2 months, 6 months, and 12 months is also calculated.

The HAQ-DI is a subscale of the ACR20/50/70. It is a completely patient-reported outcome, bridged between biochemical and physical measurements, and widely used in RA clinical trials to assess the disease activity and the patient’s disability [26]. The HAQ-DI measures eight dimensions of functional activity, including dressing, rising, eating, walking, hygiene, reach, grip, and usual activities. Each item is graded by four degrees, and scored from 0 to 3. A score 0 means without any difficulty, 1 means with some difficulty, 2 means with much difficulty, and 3 means unable to do the activity. The score in the HAQ-DI is the sum of each item averaged as an overall HAQ-DI score of 0–3. Only when at least six of the eight dimensions are answered is the scale valid. Generally, an HAQ-DI score 0–1 represents mild to moderate difficulty, 1–2 represents moderate to severe disability, and 2–3 represents severe to extreme disability [27]. Patient Assessment of Arthritis Pain and Patient Global Assessment of Arthritis are two subscales of the ACR20/50/70 and are measured by a VAS of 0–100 mm and of 0–10 cm, respectively [16].

Patients with RA often have sleep disturbance, and the more severe the disease, the more severe the sleep problems [16, 28]. The AIS, a self-assessment psychometric instrument that was designed to quantify sleep difficulty, consists of eight items, including sleep induction, awakenings during the night, final awakening, total sleep duration, sleep quality during the night, wellbeing during the night, functioning capacity during the day time, and sleepiness during the day time. Each item is scored from 0 to 3, the higher score representing poor sleep quality. The scale maximum is 24 [29].

The SF-36 consists of 36 items and measures eight dimensions, which are physical functioning, bodily pain, general health perceptions, physical role functioning, emotional role functioning, social role functioning, and mental health [16]. The SF-36 is a patient-reported survey of patient health and is widely used in the assessment of the life quality of patients with RA [16, 30]. In addition, concomitant medication is recorded as a secondary outcome.

### Safety assessments

The Juanbi pill has been used for hundreds of years in China, and the herbs in the Juanbi pill are safe according to the recommended amount in *Pharmacopoeia of the People’s Republic of China* (2015 version). Our preliminary experiment from December 2015 to May 2016 into the use of the Juanbi pill combined with MTX versus MTX alone did not show any side effects during the 3-month treatment period or in the 3-month follow-up period. We still need to perform a series of measures including subjective description and laboratory tests (especially on gastrointestinal intolerance, irritability, and kidney and liver damage) to assess the safety of the Juanbi pill during the entire trial.

At each visit, patients will be asked whether there are any adverse effects during the study period. In addition, we will perform laboratory testing of the participants’ blood, urine, feces, and kidney and liver function. We are ready to provide an appropriate treatment to the participant immediately if an adverse event is reported. Emergency services will be provided in the case of serious adverse events, and we will be prepared to report the event to the Institutional Review Board within 24 h from the time of recognition.

### Participant timeline

**Fig. 2:**
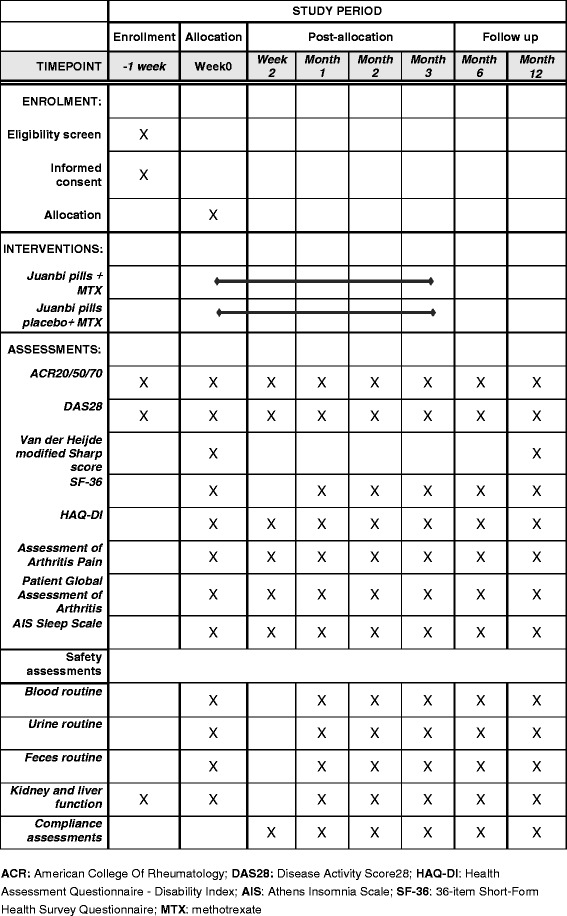
Schedule of enrollment and assessments. ACR, American College of Rheumatology; DAS28, 28-joint Disease Activity Score; HAQ-DI, Health Assessment Questionnaire-Disability Index; AIS, Athens Insomnia Scale; SF-36, 36-item Short-Form Health Survey; MTX, methotrexate

Study recruitment started in December 2016, and is expected to end in December 2017. The final follow up of all participants will end on 31 December 2018. The overview of the participant process is shown in Fig. 1, and the schedule of enrollment and assessments is provided in Fig. 2.

### Sample size calculation

We calculated the sample size according to our primary study. We conducted a preliminary experiment from December 2015 to May 2016 into the efficacy of the Juanbi pill combined with MTX versus MTX alone, and we found that patients taking the Juanbi pill combined with MTX achieved 84.8% ACR50, whereas patients in the MTX group achieved 55.5% ACR50 response. According to the formula of the rate in completely random design:

$$ \mathrm{n}1=\mathrm{n}2=\frac{\left[{u}_{\alpha /2}\sqrt{2\overline{p}\left(1-\overline{p}\right)}+{u}_{\beta}\sqrt{p_1\left(1-{p}_1\right)+{p}_2\left(1-{p}_2\right)}\right]\ }{{\left({p}_1-{p}_2\right)}^2}, $$

where n_1_ and n_2_ are the number of participants in the Juanbi and placebo groups, respectively, and *u*_*α*/2_=1.96 when type 1 error is 0.05, *u*_*β*_ = 1.282 when type II error is 0.1 in two-sided tests: $\overline{p}$ is the mean of *p*_1_ and *p*_2_ [31]. It was estimated that approximately 50 participants per group were needed to achieve 90% power and a (two-sided) 5% significance level in detecting treatment differences. Thus, the final sample size has been set at a total of 120 patients (60 in each group), considering a 20% dropout rate. Forty participants (20 in the treatment group and 20 in the placebo group) from the three centers will be recruited.

### Statistical analysis

Efficacy and safety analyses will be conducted according to the intention-to-treat principle. The method of last observation carried forward will be applied in analysis of missing values. All statistical analyses will be performed using Statistical Packages of Social Sciences software (SPSS) (version 21.0). A *p* value < 0.05 will be defined as a statistically significant result. Means and standard deviations will be used to describe the continuous variables, such as demographic and clinical outcome variables, whereas percentages will be used for categorical variables such as the rate. Continuous variables following the normal distribution will be analyzed by Student’s *t* test; otherwise, non-parametric tests will be used to compare group differences.

### Data collection and monitoring

This is a 12-month clinical trial in which participants are required to take the research medication for 3 months with follow-up visits at 6 and 12 months, attending seven assessment visits in all. Participants will receive disease activity assessment seven times (at baseline and 2 weeks, and at 1, 2, 3, 6, and 12 months), and six safety assessments (at baseline, and at 1, 2, 3, 6, and 12 months). Longhua Hospital affiliated with Shanghai University of Traditional Chinese Medicine is responsible for quality control.

### Quality control

All the pills, including the Juanbi pill and the placebo, are provided by Jiangyin Tianjiang Pharmaceutical Co., Ltd. In addition, the pharmaceutical company will conduct the randomization of participants and allocation to the three centers. Before we began the clinical trial, we carried out unified training to make sure the multicenter physicians, nurses, and outcome measurement operative involved in the trials fully understood the process of the entire trial. When the clinical trial started we set investigators to supervise the three centers every month, making sure: (1) every center has recruited the planned number of participants; (2) all the participants recruited fully meet the inclusion criteria and do not meet the exclusion criteria; and (3) all the participants have fully followed the clinical trial process, and the case report form (CRF) has been filled in as required in the recruitment and follow up. Meanwhile, we call every participant to verify the case report form and data collection are recorded in an electronic CRF monthly during the course of the clinical trial.

## Discussion

Chinese herbs have been widely used worldwide as CAM. A recent survey conducted in San Francisco showed that patients frequently used Chinese herbal products in conjunction with Western prescription medicines, which would be documented in the medical record [26]. Although many who people use Chinese herbs consider these treatments effective, there remains a need for a solid evidence to show the effectiveness and safety [27, 28].

Before we conducted this trial, we closely searched PubMed, EMBASE, the Cochrane Library, CINAHL, ISI web of knowledge, CNKI (including China Doctor/Master Dissertation Full Text Database and China Proceedings Conference Full Text Database), Vip Journal Integration Platform (VJIP), Wan Fang Data, Chinese BioMedical (CBM) databases (Sinomed), and clinical trials from the inception to December 2015, and we found no definite evidence about the effectiveness and safety of the Juanbi pill in the treatment of active RA. Therefore, we decided to conduct a multicenter, randomized, controlled clinical trial to closely study the effectiveness and safety. We will use the ACR50, DAS28, and van der Heijde modified Sharp score to estimate disease activity and damage in the affected joints; moreover, we will calculate the patients’ life quality using scales such as the HAQ-DI, AIS, and SF-36 as secondary outcome measures. We will assess the safety of the Juanbi pill in two categories: patients’ self-reporting of quality of life and symptoms, and laboratory examinations such as blood, urine, feces, and kidney and liver function tests, to obtain a full assessment. Due to lack of evidence about the effectiveness of the Juanbi pill, we will apply the pill combined with MTX to treat RA to ensure the compliance of participants and meet ethical considerations, since MTX is a first-line treatment among disease-modifying anti-rheumatic drugs, and can achieve 53.8% DAS28 remission [10, 30].

To the best of our knowledge, this is the first well-designed, randomized, controlled trial investigating the efficacy of the Juanbi pill combined with MTX for the treatment of active RA. This study is built on our preliminary open experiment with a small sample, as well as hundreds of years’ use of the Juanbi pill in China for the treatment of RA.

This study is designed to answer whether the Juanbi pill combined with MTX is preferred in the treatment of active RA, compared with MTX alone. If this trial succeeds, it will provide the patients and physicians an option of combining the Juanbi pill with MTX to obtain better disease remission. Moreover, this trial will provide data about the effect of the Juanbi pill on joint pain and function, quality of life, and safety. The results will aid clinical decision-making for the management of active RA and will provide useful information that can be incorporated into future guidelines.

### Trial status

Recruitment started in December 2016 and is expected to finish in December 2018.

## Additional file

Additional file 1: SPIRIT 2013 checklist: Recommended items to address in a clinical trial protocol and related documents. (PDF 284 kb)

## Abbreviations

ACR: American College of Rheumatology
AIS: Athens Insomnia Scale
CAM: Complementary and alternative medicine
CRP: C-reactive Protein
DAS: Disease Activity Score
ESR: Erythrocyte sedimentation rate (acute phase reactant value)
HAQ-DI: Health Assessment Questionnaire - Disability Index
MTX: Methotrexate
RA: Rheumatoid arthritis
SF-36: 36-Item Short-Form Health Survey Questionnaire
TCM: Traditional Chinese medicine
TNF: Tumor necrosis factor
VAS: Visual analogue scale
