# Supplementary material for: The efficacy of the traditional Chinese medicine Juanbi pill combined with methotrexate in active rheumatoid arthritis: study protocol for a randomized controlled trial
Source: Trials. 2018 Mar 20;19:188. doi: 10.1186/s13063-018-2555-1 (PMC5859450; doi:10.1186/s13063-018-2555-1)
Supplement: Supplementary file 1 — SPIRIT 2013 checklist: Recommended items to address in a clinical trial protocol and related documents. (PDF 284 kb) [file 13063_2018_2555_MOESM1_ESM.pdf]

SPIRIT 2013 Checklist: Recommended items to address in a clinical trial protocol and related documents*

Section/item	Item No	Description
**Administrative information**		
Title	1	Descriptive title identifying the study design, population, interventions, and, if applicable, trial acronym<br>A three-centre randomized controlled trial, 120 patients included, Juanbi Pill combined with methotrexate VS Juanbi Pill placebo combined with methotrexate.
Trial registration	2a	Trial identifier and registry name. If not yet registered, name of intended registry<br>ClinicalTrials.gov ID: NCT02885597 (registered on 30 August 2016)
	2b	All items from the World Health Organization Trial Registration Data Set<br>Not applicable.
Protocol version	3	Date and version identifier<br>November 2016, version 1.0
Funding	4	Sources and types of financial, material, and other support<br>National Natural Science Foundation (81330085, 81220108027 and 81673990), Special funding for the National Outstanding Doctoral Dissertation (201276), Key Laboratory of theory and therapy of muscles and bones, Ministry of Education（Shanghai University of Traditional Chinese Medicine）

Roles and responsibilities	5a	Names, affiliations, and roles of protocol contributors<br>Qiong Wang, Yi-Ru Wang, Qing-Yun Jia, Li Liu, Chong-Qing Xu, Xiao-Yun Wang, Min Yao, Xue-Jun Cui, Qi Shi and Qian-Qian Liang are from Department of Orthopaedics, Longhua Hospital, Shanghai University of Traditional Chinese Medicine and Institute of Spine, Shanghai University of Traditional Chinese Medicine, while Yong-Jun Wang is from Rehabilitation Medicine College, Shanghai University of Traditional Chinese Medicine. WQ and WYR is co-first author of this manuscript, contributing equally to the design, conduct the trials and draft the manuscript. All authors participated in the design of the study and performed the trial. LQQ, CXJ, SQ and WYJ supervised and coordinated the clinical trial. LL, XCQ, JQY, WXY and WYR are responsible for recruiting the participants. WQ and YM are participated in statistical design. All authors read and approved the final manuscript. LQQ and WYJ conceived of the study and revised the manuscript critically for important intellectual content.
	5b	Name and contact information for the trial sponsor<br>Qian-qian Liang, Department of Orthopaedics, Longhua Hospital, Shanghai University of Traditional Chinese Medicine, 725 South Wan-Ping Road, Shanghai 200032, China.<br>Fax: +86 216443 4704; Email: liangqianqiantcm@126.com<br>Or<br>Yong-jun Wang, Institute of Spine, Shanghai University of Traditional Chinese Medicine, 725 Wan-Ping South Road, Shanghai 200032, China.<br>Fax: +86 216443 4704; Email: yjwang8888@126.com
	5c	Role of study sponsor and funders, if any, in study design; collection, management, analysis, and interpretation of data; writing of the report; and the decision to submit the report for publication, including whether they will have ultimate authority over any of these activities<br>LQQ and WYJ supervised and coordinated the clinical trial, conceived of the study and revised the manuscript critically for important intellectual content.
	5d	Composition, roles, and responsibilities of the coordinating centre, steering committee, endpoint adjudication committee, data management team, and other individuals or groups overseeing the trial, if applicable (see Item 21a for data monitoring committee)<br>Longhua Hospital affiliated to Shanghai university of Traditional Chinese Medicine, Yueyang Integrated Medicine Hospital affiliated to Shanghai university of Traditional Chinese Medicine, and Shanghai Guanghua Hospital of Integrated Traditional Chinese and Western Medicine.

Introduction

Background and rationale	6a	Description of research question and justification for undertaking the trial, including summary of relevant studies (published and unpublished) examining benefits and harms for each intervention<br>To observe the effect of Juanbi pill combined with methotrexate on the treatment of active RA and its side effects.<br>Methotrexate is the first line medicine on the treatment of RA and Juanbi Pill have been used in Chinese for thousands of years to manage active RA with no detectable side effect, nevertheless, there is no solid evidence to show the effect of Juanbi pill for the management of active RA.
	6b	Explanation for choice of comparators<br>The comparator is Juanbi Pill placebo and methotrexate for methotrexate is the first line medicine on the treatment of RA
Objectives	7	Specific objectives or hypotheses<br>Juanbi Pill combined methotrexate is preferred in the treatment of active RA, compared to MTX alone.
Trial design	8	Description of trial design including type of trial (eg, parallel group, crossover, factorial, single group), allocation ratio, and framework (eg, superiority, equivalence, noninferiority, exploratory)<br>Two arms parallel group, 1:1, superiority

Methods: Participants, interventions, and outcomes

Study setting	9	Description of study settings (eg, community clinic, academic hospital) and list of countries where data will be collected. Reference to where list of study sites can be obtained<br>Longhua Hospital affiliated to Shanghai university of Traditional Chinese Medicine, Yueyang Integrated Medicine Hospital affiliated to Shanghai university of Traditional Chinese Medicine, and Shanghai Guanghua Hospital of Integrated Traditional Chinese and Western Medicine. All of the hospital listed above is in Shanghai, China.

Eligibility criteria	10	Inclusion and exclusion criteria for participants. If applicable, eligibility criteria for study centres and individuals who will perform the interventions (eg, surgeons, psychotherapists)<br>Participants should be :1)adults (aged≥18 years old) with rheumatoid arthritis, 2) satisfies ACR (American College Of heumatology)/EULAR (European League Against Rheumatism) criteria for RA, 2010, 3)have an onset of symptoms within 12 months before enrollment, 4) have an active disease activity at the time of enrollment as indicated by DAS28 score more than 3.2, no prior exposure to more than 10 mg oral glucocorticoids daily or any biologic agents, 5) paid employment or unpaid but measurable work (e.g., caring for a family and home).<br>Participants should not be: 1)combined with other auto-immune disease such as adjuvant arthritis, lupus arthritis, osteoarthritis and et al., 2)abnormal liver and my kidney function, 3) pregnancy or have a plan of pregnancy, breast feeding women, 4)severe chronic or acute disease interfering with therapy attendance, 5)alcohol or substance abuser 6)unable to understand or sign an informed consent form.<br>If applicable, eligibility criteria for study centres and individuals a physician will perform the interventions
Interventions	11a	Interventions for each group with sufficient detail to allow replication, including how and when they will be administered<br>Patients in intervention group will administrate Juanbi pill (4 g) in 200 milliliter hot water as the instruction and take the solution orally twice a day for 3 months. While patients in the placebo group will take Juanbi pill placebo as the same way as the Juanbi pill group.
	11b	Criteria for discontinuing or modifying allocated interventions for a given trial participant (eg, drug dose change in response to harms, participant request, or improving/worsening disease)<br>When serious adverse effects occur, we will provide an appropriate treatment to the subject immediately and record the adverse effect and stop the subject to continue to take the given medicine.
	11c	Strategies to improve adherence to intervention protocols, and any procedures for monitoring adherence (eg, drug tablet return, laboratory tests)<br>We will call the patients to do laboratory tests according to the schedule and ask them to record the medicine they took carefully in a note book.
	11d	Relevant concomitant care and interventions that are permitted or prohibited during the trial<br>Patients are not allowed to use other medicine excepted the given medicine except less than 5mg a day oral glucocorticoid in principle.

Outcomes	12	Primary, secondary, and other outcomes, including the specific measurement variable (eg, systolic blood pressure), analysis metric (eg, change from baseline, final value, time to event), method of aggregation (eg, median, proportion), and time point for each outcome. Explanation of the clinical relevance of chosen efficacy and harm outcomes is strongly recommended<br>Primary outcomes: 1) the change rate of ACR50, changes of The Disease Activity Score (DAS) 28 from the baseline to 3 months<br>2) change score of van der Heijde modified Sharp from the baseline to 12 month follow-up.<br>ACR50 are more desirable targets for patients and provide useful information. DAS28 is widely used as an indicator of RA disease activity and response to treatment and widely used in clinical trial to assess the treatment effect.<br>Van der Heijde modified Sharp score can precisely reflect the bone condition of RA patients.<br>The ACR 50 and DAS 28 will be estimated at the baseline, 1 month, 2 months, 3 months, 4 time points during the treatment period and 6 months and 12 months follow-up period. While the Van der Heijde modified Sharp will be measured only at the baseline and 12 months follow-up.<br>ACR 50 will be described in proportion while DAS28 and van der Heijde modified Sharp will be in mean ±SD<br>The secondary outcome will be to compare the change rate of ACR20/70, HAQ-DI, Patient Assessment of Arthritis Pain, Patient Global Assessment of Arthritis, and AIS Sleep Scale from baseline to 2 weeks, 1 month, 2 month, 3months, 6 months and 12 months follow-ups. The change rate of ACR50 and DAS28 from the baseline to 2 weeks, 1 month, 2 month, 6 months and 12 months follow-ups also belongs to the secondary outcome measures, while the change score of 36-item Short-Form Health Survey Questionnaire (SF36) form the baseline to 1 month, 2 month, 6 months and 12 months also calculated.<br>HAQ-DI is one subscale of ACR20/50/70. It's a completely patient-reported outcome, bridged between biochemical and physical measurements, and widely used in RA clinical trials to assess the disease activity and patient's disability.<br>Patient Assessment of Arthritis Pain and Patient Global Assessment of Arthritis are 2 subscales of ACR20/50/70 and measured by a Visual Analogue Scale (VAS) of 0-100 mm and 0-10 respectively. Patients suffering from RA often has sleep disturbance, the severer the disease, the poorer sleep the patients will suffer (10, 22). Athens Insomnia Scale (AIS) a self-assessment psychometric instrument designed to quantify sleep difficulty.<br>SF-36 is widely used in the assessment of RA patients' life quality. All the second outcome are measure by mean ±SD except ACR20/50/70 by proportion.<br>In addition, concomitant medication also recorded as a secondary outcome, which will be described by description.

Participant timeline	13	Time schedule of enrolment, interventions (including any run-ins and washouts), assessments, and visits for participants. A schematic diagram is highly recommended (see Figure)<br>Table 1 in the manuscript
Sample size	14	Estimated number of participants needed to achieve study objectives and how it was determined, including clinical and statistical assumptions supporting any sample size calculations<br>We calculate the sample size according to our primary study. We conducted a preliminary experiment about Juanbi Pill combined with MTX VS MTX between December 2015 and May 2016, we found that Juanbi Pill combined with MTX could achieve ACR 50 84.8% while the MTX group only 55.5%. The formula of the rate in completely random design is n1=n2=([u_(α/2) √(2p ̅(1-p ̅))+u_β √(p_1 (1-p_1)+p_2 (1-p_2))])/ 〖(p_1-p_2)〗^2 , among which, n1 and n2 are the number of Juanbi and the placebo group, u_(α/2)=1.96 when type 1 error is 0.05, u_β=1.282 when type II error is 0.1 in two-sided tests. p ̅is the mean of p_1 and p_2 . We estimated that approximately 50 participants each group were needed to achieve 90% power and a (two-sided) 5% significance level in detecting treatment differences. We estimated that a total of 120 patients (60 for each group) would be recruited to ensure statistically significant results, considering 20% droupout rate.
Recruitment	15	Strategies for achieving adequate participant enrolment to reach target sample size<br>By poster in hospital and hospital official website advertisement

Methods: Assignment of interventions (for controlled trials)

Allocation:

Sequence generation	16a	Method of generating the allocation sequence (eg, computer-generated random numbers), and list of any factors for stratification. To reduce predictability of a random sequence, details of any planned restriction (eg, blocking) should be provided in a separate document that is unavailable to those who enrol participants or assign interventions<br>A random number list will be generated by Excel (office 2016, Microsoft Corporation) to either the Juanbi Pill group or the placebo group randomly in a 1:1 ratio.

Allocation concealment mechanism	16b	Mechanism of implementing the allocation sequence (eg, central telephone; sequentially numbered, opaque, sealed envelopes), describing any steps to conceal the sequence until interventions are assigned<br>When a participant is recruited in, the investigator will provide the pharmaceutical factory a number, the pharmaceutical factory will randomly post the Pill (or the placebo) to the participant according to the random number list. The provided number and the corresponding number list will be recorded and will be kept in a locked cabinet in the locked office of the pharmaceutical factory.
Implementation	16c	Who will generate the allocation sequence, who will enrol participants, and who will assign participants to interventions<br>The pharmaceutical factory will generate the allocation sequence, the care providers will enrol participants and The pharmaceutical factory will assign participants to interventions.
Blinding (masking)	17a	Who will be blinded after assignment to interventions (eg, trial participants, care providers, outcome assessors, data analysts), and how<br>The stuff in the pharmaceutical factory do not participant in the trials, and the investigator, doctors, nurses, outcome measuring person, statisticians and the participants have no idea about the group information until the end of the trial, when all statistics work are finished.
	17b	If blinded, circumstances under which unblinding is permissible, and procedure for revealing a participant's allocated intervention during the trial<br>Sever sided effect occurred. We will report the event to the Institutional Review Board within 24 hours from the time of recognition at the same time as required. We will ask the pharmaceutical factory to tell the participant who had sever sided effect which group he/she is in by adequate procedure.

Methods: Data collection, management, and analysis

Data collection methods	18a	Plans for assessment and collection of outcome, baseline, and other trial data, including any related processes to promote data quality (eg, duplicate measurements, training of assessors) and a description of study instruments (eg, questionnaires, laboratory tests) along with their reliability and validity, if known. Reference to where data collection forms can be found, if not in the protocol<br>Training of assessors in the assessment and collection of data. In addition, we had made a questionnaires to guide the assessors in detail.

	18b	Plans to promote participant retention and complete follow-up, including list of any outcome data to be collected for participants who discontinue or deviate from intervention protocols<br>Efficacy and safety analyses will be conducted according to the intention-to-treat principle. Last-observation-carried-forward method will be applied in the missing values.
Data management	19	Plans for data entry, coding, security, and storage, including any related processes to promote data quality (eg, double data entry; range checks for data values). Reference to where details of data management procedures can be found, if not in the protocol<br>Epidata (version 3.0) procedure will be used to restrict data values and we will adopt double data entry to promote data quality.
Statistical methods	20a	Statistical methods for analysing primary and secondary outcomes. Reference to where other details of the statistical analysis plan can be found, if not in the protocol<br>All statistical analyses will be performed using Statistical Packages of Social Sciences software (SPSS) (version 21.0). P-value <0.05 will be defined as statistical significance. Means and standard deviations will be described in the continuous variable such as demographic, clinical, outcome variables clinical and et al. while the percentages will be used for categorical variables such as the rate. Continuous variable followed the normal distribution will be calculated by Student's t-tests, otherwise non-parametric test will be used to compare group differences.
	20b	Methods for any additional analyses (eg, subgroup and adjusted analyses)<br>A subgroup analyses will be conducted when necessary.
	20c	Definition of analysis population relating to protocol non-adherence (eg, as randomised analysis), and any statistical methods to handle missing data (eg, multiple imputation)<br>We will adopt multiple imputation to handle missing data statistically.
**Methods: Monitoring**		
Data monitoring	21a	Composition of data monitoring committee (DMC); summary of its role and reporting structure; statement of whether it is independent from the sponsor and competing interests; and reference to where further details about its charter can be found, if not in the protocol. Alternatively, an explanation of why a DMC is not needed<br>Longhua Hospital affiliated to Shanghai university of Traditional Chinese Medicine is responsible for quality control. The DMC is in charge of data entry, coding, security, and storage. There are five employee in the DMC and they are independent from the sponsor and there are no competing interests, which can be found http://www.longhua.net/ywsy/gzlc/287.jhtml

	21b	Description of any interim analyses and stopping guidelines, including who will have access to these interim results and make the final decision to terminate the trial<br>Not applicable.
Harms	22	Plans for collecting, assessing, reporting, and managing solicited and spontaneously reported adverse events and other unintended effects of trial interventions or trial conduct<br>At each visit, patients will be asked whether there are any adverse effects during the study period. When an adverse event was claimed, we will provide an appropriate treatment to the subject immediately and record the adverse effect. An emergency services will be provided in case of serious adverse events. In addition, we will test the patients' blood routine, urine routine, feces routine, kidney and liver function.
Auditing	23	Frequency and procedures for auditing trial conduct, if any, and whether the process will be independent from investigators and the sponsor<br>Half a year and the process will be independent from investigators and the sponsor.

Ethics and dissemination

Research ethics approval	24	Plans for seeking research ethics committee/institutional review board (REC/IRB) approval<br>The trial protocol has been approved by the Research Ethical Committee of Longhua hosptial, affiliated to Shanghai University of Traditional Chinese Medicine, Shanghai, China (approval Number 2016LCSY033), and we have got the oral permission of the other 2 centres and we will got formal approval number in this month.
Protocol amendments	25	Plans for communicating important protocol modifications (eg, changes to eligibility criteria, outcomes, analyses) to relevant parties (eg, investigators, REC/IRBs, trial participants, trial registries, journals, regulators)<br>NO
Consent or assent	26a	Who will obtain informed consent or assent from potential trial participants or authorised surrogates, and how (see Item 32)<br>The care provider will invite the patient to participate the trial, tell them in detail why we should take this trial and what kind of rights, obligations and risks they will have if they participate the trials. And the care provider will give them a written informed consent. Only the patients fully understand and sign the informed consent, can they participant the trial.
	26b	Additional consent provisions for collection and use of participant data and biological specimens in ancillary studies, if applicable<br>Not applicable.

Confidentiality	27	How personal information about potential and enrolled participants will be collected, shared, and maintained in order to protect confidentiality before, during, and after the trial<br>The personal information about the potential and enrolled participants will be collected to be used **only** in this trial, and we won't share or maintained the personal information when unnecessary.
Declaration of interests	28	Financial and other competing interests for principal investigators for the overall trial and each study site<br>We declared there were no competing interests for principal investigators for the overall trial and each study site
Access to data	29	Statement of who will have access to the final trial dataset, and disclosure of contractual agreements that limit such access for investigators<br>Statisticians.
Ancillary and post-trial care	30	Provisions, if any, for ancillary and post-trial care, and for compensation to those who suffer harm from trial participation<br>We will give a certain amount of provision according to Institutional Review Board when necessary.
Dissemination policy	31a	Plans for investigators and sponsor to communicate trial results to participants, healthcare professionals, the public, and other relevant groups (eg, via publication, reporting in results databases, or other data sharing arrangements), including any publication restrictions<br>We had no planes.
	31b	Authorship eligibility guidelines and any intended use of professional writers<br>We had no planes.
	31c	Plans, if any, for granting public access to the full protocol, participant-level dataset, and statistical code<br>We had no planes.
**Appendices**		
Informed consent materials	32	Model consent form and other related documentation given to participants and authorised surrogates<br>The consent materials had been approved by the Research Ethical Committee of Longhua hospital, and we had attached them to supplementary materials.
Biological specimens	33	Plans for collection, laboratory evaluation, and storage of biological specimens for genetic or molecular analysis in the current trial and for future use in ancillary studies, if applicable<br>Not applicable.

*It is strongly recommended that this checklist be read in conjunction with the SPIRIT 2013 Explanation & Elaboration for important clarification on the items. Amendments to the protocol should be tracked and dated. The SPIRIT checklist is copyrighted by the SPIRIT

Group under the Creative Commons "Attribution-NonCommercial-NoDerivs 3.0 Unported" license.
